# Sex-Dependent Alterations of Regional Homogeneity in Cigarette Smokers

**DOI:** 10.3389/fpsyt.2022.874893

**Published:** 2022-04-25

**Authors:** Zhi Wen, Xu Han, Yao Wang, Weina Ding, Yawen Sun, Yan Kang, Yan Zhou, Hao Lei, Fuchun Lin

**Affiliations:** ^1^Department of Radiology, Renmin Hospital of Wuhan University, Wuhan, China; ^2^Department of Radiology, Ren Ji Hospital, School of Medicine, Shanghai Jiao Tong University, Shanghai, China; ^3^State Key Laboratory of Magnetic Resonance and Atomic and Molecular Physics, National Center for Magnetic Resonance in Wuhan, Wuhan Institute of Physics and Mathematics, Innovation Academy for Precision Measurement Science and Technology, Chinese Academy of Sciences, Wuhan, China; ^4^University of Chinese Academy of Sciences, Beijing, China

**Keywords:** sex, regional homogeneity, resting-state fMRI, craving, smokers

## Abstract

Biological sex may play a large role in cigarette use and cessation outcomes and neuroimaging studies have demonstrated that cigarette smoking is associated with sex-related differences in brain structure and function. However, less is known about sex-specific alterations in spontaneous brain activity in cigarette smokers. In this study, we investigated the sex-related effects of cigarette smoking on local spontaneous brain activity using regional homogeneity (ReHo) based on resting-state fMRI. Fifty-six smokers (24 females) and sixty-three (25 females) healthy non-smoking controls were recruited. Whole-brain voxelwise 2-way analysis of covariance of ReHo was performed to detect brain regions with sex-dependent alterations on the spontaneous brain activity. Compared to non-smokers, smokers exhibited significant ReHo differences in several brain regions, including the right medial orbitofrontal cortex extended to the ventral striatum/amygdala/parahippocampus, left precuneus, and bilateral cerebellum crus. Smoking and sex interaction analysis revealed that male smokers showed significantly lower ReHo in the right ventral striatum, left cerebellum crus1, and left fusiform gyrus compared to male non-smokers, whereas there are no significant differences between female smokers and non-smokers. Furthermore, the ReHo within the left cerebellum crus1 was negatively correlated with craving scores in male smokers but not in female smokers. Such sex-dependent differences in spontaneous brain activity lays a foundation for further understanding the neural pathophysiology of sex-specific effects of nicotine addiction and promoting more effective health management of quitting smoking.

## Introduction

Tobacco smoking, the leading cause of preventable death worldwide, is associated with many serious health problems including cardiovascular disease and lung cancer (1). Despite the growing number of evidence-based treatments for smoking addiction, the efficacy remains moderate, with high relapse rate (2). Biological sex may play a large role in patterns of nicotine use and cessation. outcomes For instance, higher smoking rates, greater reinforcement of nicotine and better nicotine replacement therapies has been reported among men than women (3). Women are inclined to smoke for stress relief and mood regulation, and difficult to maintain abstinence (4). Therefore, sex differences may be crucial to target the neural pathophysiology of nicotine addiction and develop more effective relapse prevention therapies (5).

Neuroimaging studies showed that cigarette smoking is associated with sex-specific alterations in brain structure and function. Compared to sex-matched non-smokers, male rather than female nicotine-dependent subjects had a larger volume in the left putamen and a smaller volume in the left caudate, whereas female but not male smokers showed a smaller volume in the right amygdala (6). Exposure to smoking cues, direct comparisons between male and female smokers revealed that male smokers showed greater reactivity in reward-related brain regions (7) and greater coupling between the interhemispheric regions within the executive control network (8) than female smokers. In contrast, female smokers had greater connectivity within the limbic and default mode network than male smokers (9, 10). Earlier work of our team analyzed middle-aged chronic heavy smokers and found that the regional homogeneity (ReHo) of the right paracentral lobule of male smokers was significantly different from that of female smokers (11). Another study of our team reported that young male but not young female smokers had decreased resting-state functional connectivity (rsFC) between the right amygdala and right orbitofrontal cortex (OFC) (12). RsFC of the amygdala-OFC circuity was negatively correlated with the craving score in male but not female smokers (12). Despite increasing knowledge of the sex-specific effects of tobacco use on brain structure and connectivity between spatially distinct brain regions, it remains unclear whether cigarette smoking has differential influence on the local spontaneous brain activity between men and women, particularly young adults.

Resting-state fMRI (rs-fMRI) provides a non-invasive method of assessing changes in resting-state brain activity and functional connectivity across the whole-brain (13). ReHo measures the synchronization of intra-regional spontaneous low-frequency BOLD signal fluctuations by calculating Kendall's coefficient of concordance (14), which have high test-retest reliability (15). The ReHo metric has been widely used to investigate abnormal local spontaneous brain activity in psychiatric and neurologic conditions (16). Recent literature review demonstrated differences in adolescent vulnerability to neurotoxicity of drugs of abuse from adult (17). Brain imaging studies showed humans might achieve full adulthood after the ages of 25 and 30 (18, 19). Hence, it is important to study the abnormal changes of brain function in smokers at younger age group. In view of the gender influence of smoking on neural structure and functional networks, we assume that young male and female smokers would show constant change patterns in the reward-related areas [as in our previous article (11)], and display different change patterns in local synchronization of the brain.

To test our hypothesis, we employed ReHo method to depict spontaneous brain activity and then performed a voxel-wise analysis to examine the intrinsic brain activity differences between smokers and non-smokers, and to further explore the sex interaction on smoking dependency. Such sex-dependent differences in spontaneous brain activity lays a foundation for further understanding the neural pathophysiology nicotine addiction and promoting more effective health management of quitting smoking.

## Materials and Methods

### Subjects

Sixty cigarette smokers (25 females) and 67 healthy nonsmoking controls (28 females) aged 18–29 years participated in this study. The Mini International Neuropsychiatric Interview was used to exclude participants who had substance use disorder other than nicotine dependence, current Axis 1 DSM V psychiatric diagnoses, or significant medical conditions. Exclusion criteria for both smokers and non-smokers were a history of head trauma or injury causing loss of consciousness lasting >3 min or associated with skull fracture or intracranial bleeding, or had irremovable magnetically active objects on or within their body. Smokers were defined as those who smoked at least 10 cigarettes/day on any given day during the last year. The severity of nicotine addiction was determined from the Fagerström Test for Nicotine Dependence (FTND) (20), and the measurement of “craving” for cigarettes was evaluated using a brief questionnaire of smoking urges (21). All smokers had no period of smoking abstinence longer than 3 months in the past year. It was about 1 h since last cigarette before smokers were scanned. Non-smokers had not smoked more than five cigarettes in their lifetimes. All participants were administered a set of questionnaires at the beginning of the study, namely, the Self-rating Anxiety Scale (SAS), Self-rating Depression Scale (SDS) and Barratt Impulsiveness Scale (BIS) version 11. The dataset of subjects were described in our previous article (12), but the analyses of this study are different from that in previous reports; here we focus on exploring sex-specific effects of cigarette smoking on spontaneous brain activity.

The current study adhered to the Declaration of Helsinki and was approved by the Research Ethics Committee of Renji Hospital, School of Medicine of Shanghai Jiaotong University. All participants were informed of the measurements and the experimental procedures of the study before their MRI examinations. Each participant provided written informed consent.

### Data Acquisition

Images were obtained using a 3.0-T GE Signa HDx (Milwaukee, WI, USA) scanner with a standard 8-channel head coil. Restraining foam pads were used to reduce head motion, and earplugs were used to reduce scanner noise. Before MRI scanning, all subjects were instructed to relax with their eyes closed while refraining from falling asleep and without engaging in any directed, systematic thought. The physiological state of the smokers when they were in the scanner as spontaneous, rather than abstinence or satiety, that was, none of the smokers felt the acute urge to smoke or experience any withdrawal symptoms confirmed by a self-report completed by each participant immediately after the scan.

Rs-fMRI data were acquired using a gradient-echo echo-planar imaging sequence (TR = 2,000 ms, TE = 24 ms, flip angle = 90°, matrix = 64 × 64, FOV = 230 × 230 mm^2^, thickness = 4 mm with no gap, 34 slices). For each participant, the rs-fMRI scan lasted 7 min and 20 s and a total of 220 volumes were acquired. The axial T1-weighted and T2-weighted images were also performed to confirm the absence of structural lesions. All images were evaluated by two experienced neuroradiologists and no participants were excluded on this basis.

### Image Preprocessing

Rs-fMRI data were preprocessed using the Data Processing Assistant for Resting-State fMRI (http://rfmri.org/DPARSF). After removing the first 10 volumes for each subject, the remaining 210 volumes were corrected for slice timing and realigned to the first volume for head-motion correction. The Friston 24-parameter model (i.e., six head motion parameters, six head motion parameters one time point before, and 12 corresponding squared items) was performed to regress out the head motion effects. The scrubbing strategy was further carried out to correct the head motion effects. Time points with framewise displacement larger than 0.2 mm were identified and removed along with 1 back and 2 forward neighbors (22). Subjects with <90 time points after scrubbing were not used because too few remaining time points may lead to unreliable results (23). As a result, four smokers and four non-smokers were discarded, and a total of 56 smokers (24 females) and 63 non-smokers (25 females) were ultimately used in the ReHo analysis. The detailed demographic and clinical data of the subjects used in this study are provided in Table 1. Smokers and non-smokers were not statistically different in terms of time points removed (28.08 ± 32.07 for smokers, 36.25 ± 32.66 for non-smokers, *p* = 0.17). Afterward, the functional images were normalized to the standard Montreal Neurological Institute (MNI) space and resampled to a 3-mm isotropic voxel. Then, linear and quadratic trends as well as white matter and corticospinal fluid signals were removed. Finally, temporal bandpass filtering (0.01–0.1 Hz) was performed to reduce the effects of high-frequency physiological noise.

**Table 1:** Demographic information of the subjects in each group and between-group comparisons.

	**Smokers**		**Non-smokers**		**Group comparisons^a^ (F/*****p*** **values)**		
	**Male (*n* = 32)**	**Female (*n* = 24)**	**Male (*n* = 38)**	**Female (*n* = 25)**	**Group**	**Sex**	Group*sex
Age	22.63 ± 2.70	24.50 ± 2.95	22.58 ± 3.12	24.22 ± 3.41	0.09/0.77	9.58/0.002	0.05/0.83
Education level	13.00 ± 2.03	11.29 ± 2.48	13.82 ± 3.31	16.60 ± 4.23	28.04/ <0.001	0.87/0.35	15.09/ <0.001
Age at first smoking	16.84 ± 2.47	20.00 ± 2.86	**–**	**–**	**–**	**–**	**–**
Duration of smoking	5.78 ± 2.84	4.50 ± 2.50	**–**	**–**	**–**	**–**	**–**
FTND score	6.53 ± 2.06	6.04 ± 2.48	**–**	**–**	**–**	**–**	**–**
Craving score	30.94 ± 11.92	30.38 ± 16.14	**–**	**–**	**–**	**–**	**–**
SAS score	45.03 ± 9.39	49.83 ± 10.05	38.95 ± 6.28	40.08 ± 8.78	24.76/ <0.001	3.48/0.07	1.33/0.25
SDS score	47.91 ± 9.61	53.42 ± 9.89	42.84 ± 8.67	44.82 ± 9.00	15.71/ <0.001	4.68/0.03	1.06/0.31
BIS score	52.72 ± 7.79	60.67 ± 10.81	51.61 ± 6.28	52.68 ± 7.70	9.17/0.003	9.02/0.003	5.23/0.02

a *Analysis of variance, df = 1,115*.

*Values are expressed as the means ± standard deviations. The age, educational level, age at first cigarette and duration of smoking are displayed in years. FTND, Fagerström Test of Nicotine Dependence; SAS, Self-rating Anxiety Scale; SDS, Self-rating Depression Scale; BIS, Barratt Impulsiveness Scale version 11. Educational level was defined as the number of years of scholarship since primary school*.

### ReHo Analysis

Based on the temporally bandpass-filtered data, the ReHo value for each voxel was defined as the KCC of the time series of a given voxel and those of its 26 nearest neighbors. For standardization purposes, each individual ReHo map was divided by its mean ReHo within a brain mask without non-brain tissue (14). Finally, the ReHo maps were smoothed by 6 mm full width at half maximum Gaussian kernel.

### Statistical Analysis

A 2-way analysis of variance (ANOVA) was used to identify differences among groups in age, education level, SAS, SDS and BIS scores. A voxelwise 2-way analysis of covariance (ANCOVA) with age and education level as covariates was performed to evaluate the main effects of group (smokers vs. non-smokers) and sex (male vs. female) as well as the interaction between group and sex. A false discovery rate (FDR) corrected *p*-value of 0.05 was used for multiple comparisons. When the group effects occurred, we further explore the specific smoking effects (i.e., smokers > non-smokers or smokers < non-smokers) on ReHo. We extracted the averaged ReHo from significant clusters showing group effects, and then performed the 1-way ANCOVA controlling for age and education level to determine the specific smoking effects on ReHo. When the group-by-sex interaction effects occurred, the sex-specific effects of smoking on ReHo were investigated. The mean ReHo values from clusters showing group-by-sex interaction effects were extracted, and then the 1-way ANCOVAs controlling for age and education level were conducted to compare male smokers vs. male non-smokers and female smokers vs. female non-smokers separately.

For smokers, Pearson correlation analysis was performed to investigate whether there were correlations between the altered ReHo and the smoking-related variables (i.e., duration of smoking, age at first smoking, FTND and craving scores). A *p*-value of < 0.05 was considered significant.

## Results

### Demographic and Clinical Measures of Participants

Table 1 lists the demographic and clinical measures of the subjects used in this study. No significant difference was found in gender among the groups. For age, no main effect of group (*p* = 0.77) or group by sex interaction (*p* = 0.83) were found, while there was a significant main effect of sex (*p* = 0.002). For years of education, significant main effect of group (*p* < 0.001) and group by sex interaction (*p* < 0.001) were revealed, while there was no main effect of sex (*p* = 0.35). Specifically, male smokers had higher education level than female smokers (*p* = 0.006), while male non-smokers had lower education level than female non-smokers (*p* = 0.005).

There were no significant differences in duration of smoking (*p* = 0.09), FTND (*p* = 0.42) or craving scores (*p* = 0.88) between male and female smokers, while male smokers had an earlier age at first smoking (*p* < 0.001) than female smokers. Smokers as a whole had higher scores than non-smokers on SAS (*p* < 0.001), SDS (*p* < 0.001) and BIS (*p* = 0.003), which is consistent with prior studies showing that smoking has often been associated with increased depression, anxiety and impulsivity (24, 25). Women had higher scores on SAS (*p* = 0.07), SDS (*p* = 0.03) and BIS (*p* = 0.003) than men. Although the SAS, SDS and BIS scores of smokers were higher than those of non-smokers, they did not meet the criteria for comorbid mood or attention disorders.

### ReHo Changes in Smokers

**Figure 1:**
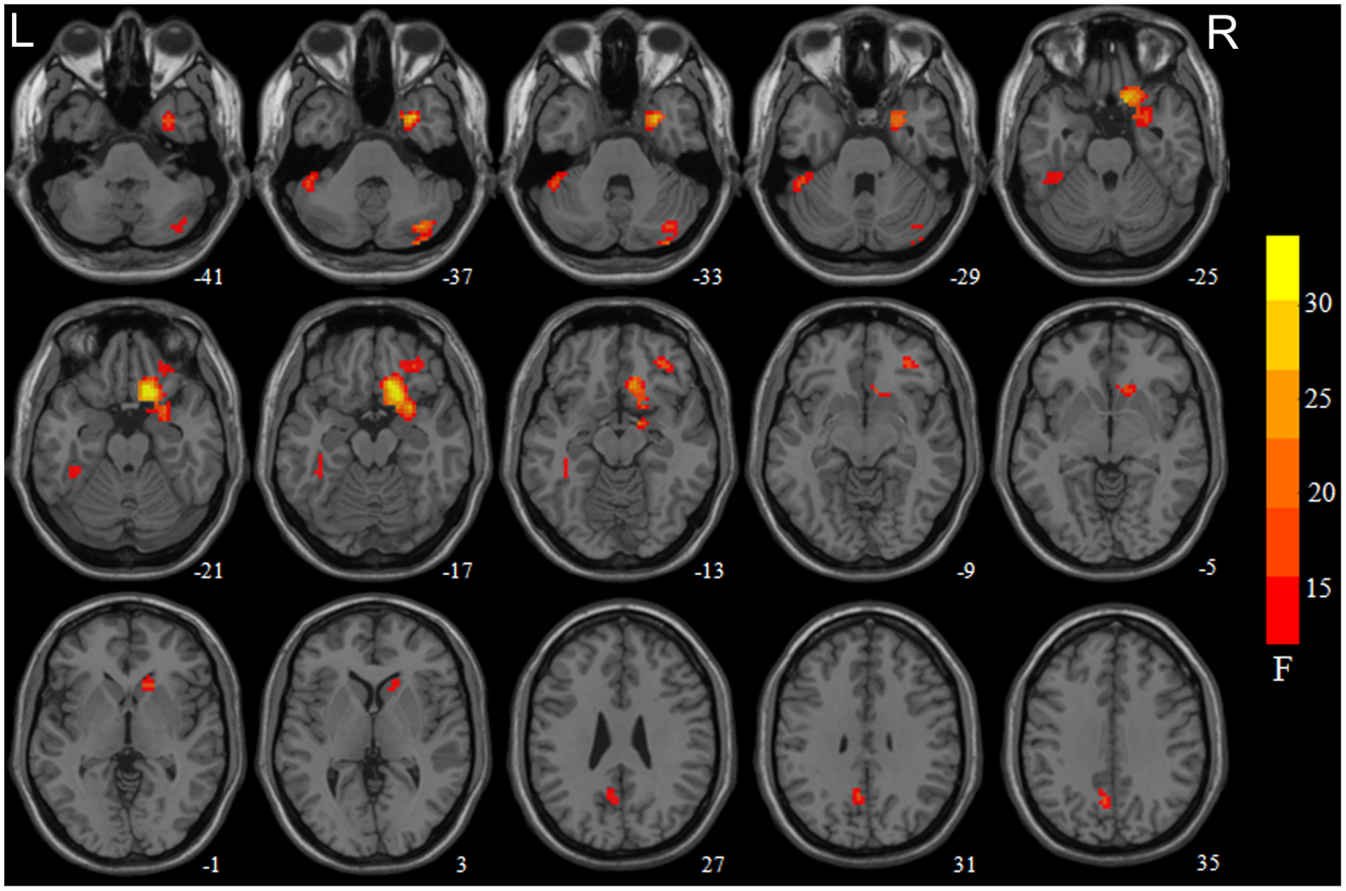
Brain areas with significant main effect of smoking on regional homogeneity (ReHo) revealed by the 2-way analysis of covariance with age and education levels as covariates. Regions in red-yellow are brain areas where ReHo was significantly altered in smokers relative non-smokers. The areas include the right medial orbitofrontal cortex (OFC) extend to the ventral striatum/amygdala/parahippocampa gyrus, right cerebellum crus1/2, left precuneus, left cerebellum crus1 extended to the inferior temporal gyrus/fusiform gyrus, and right lateral OFC. The results were multiple compared at voxel-level *p* < 0.05 (FDR corrected). The left side of the image corresponds to the left hemisphere of the brain.

Statistically significant main effects of smoking were observed for ReHo (voxel-level *p* < 0.05 FDR corrected; Figure 1). Further 1-way ANCOVA with age and education as covariates demonstrated that compared to non-smokers, smokers had significant greater ReHo in the left precuneus and right cerebellum posterior lobe (crus1/2) while lower ReHo in the right medial OFC extending to the ventral striatum/amygdala/parahippocampal gyrus, right lateral OFC, and left cerebellum anterior lobe (crus1) extending to the inferior temporal gyrus/fusiform gyrus (Table 2).

**Table 2:** Brain regions showing significant main effect of smoking on regional homogeneity (ReHo) (voxel-level *p* < 0.05 corrected for false discovery rate).

**Specific effects**	**Identified brain regions**	**Peak coordinates (MNI)**			**Side**	**Peak F**	**Cluster size (voxels)**
		***X***	***Y***	***Z***			
**Smokers** **>** **non-smokers**							
fSM > fNS, mSM > mNS	Precuneus	−6	−63	42	L	19.74	76
mSM>mNS	Cerebellum posterior lobe (Crus1, Crus2)	36	−75	−39	R	24.02	73
**Smokers** **<** **non-smokers**							
mSM < mNS	Medial orbital frontal cortex/Ventral striatum/amygdala/parahippocampal gyrus	15	21	−21	R	33.94	398
mSM < mNS	Cerebellum anterior lobe (Crus1)/inferior temporal gyrus/fusiform gyrus	−48	−42	−36	L	18.68	103
mSM < mNS	Lateral orbital frontal cortex	33	39	−12	R	18.13	61

*MNI, Montreal Neurological Institute; fSM, female smokers; fNS, female non-smokers; mSM, male smokers; mNS, male non-smokers; R, right; L, left*.

At a second stage, we analyzed the simple effects of smoking in the females and males in a separate analysis within the brain regions showing main effects of smoking. The male smokers were compared with male non-smokers and female smokers with female non-smokers. Independent 1-way ANCOVA controlling for age and education revealed that the main effects of smoking mostly came from male smokers except that female smokers had significantly higher ReHo as compared to female non-smokers in the left precuneus (for details, see Table 2).

**Figure 2:**
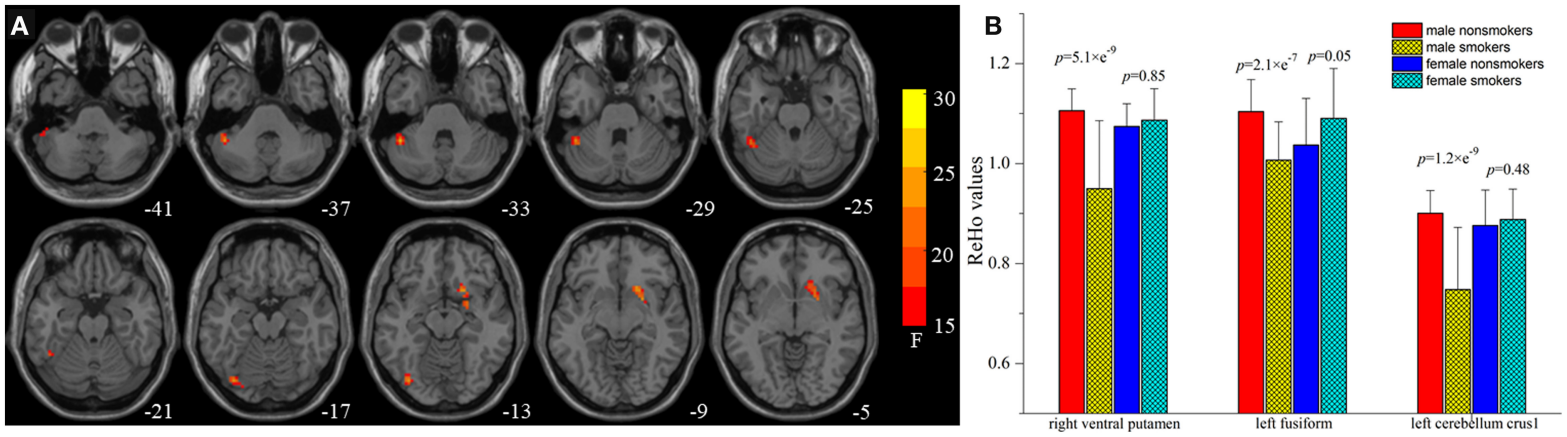
Brain areas with interaction between smoking and sex on regional homogeneity (ReHo) by the 2-way analysis of covariance (ANCOVA) with age and education levels as covariates. **(A)** Brain regions showing smoking-sex interaction on ReHo include the left fusiform gyrus, left cerebellum crus1 extended to the inferior temporal gyrus, and right ventral striatum [voxel-level *p* < 0.05 (FDR corrected]. **(B)** *Post-hoc* 1-way ANCOVA analyses revealed that male smokers had lower ReHo than male non-smokers in the right ventral striatum (*p* = 5.1 × e^−9^), left fusiform gyrus (*p* = 2.1 × e^−7^), left cerebellum crus1 extended to the inferior temporal gyrus (*p* = 1.2 × e^−9^), whereas female smokers showed no but no differences on ReHo values in these regions.

A significant smoking and sex interaction effect on ReHo was identified in the left fusiform gyrus, left cerebellum crus1 extending to the inferior temporal gyrus, and right ventral striatum/putamen (voxel-level *p* < 0.05 FDR corrected, Table 3; Figure 2A). *Post-hoc* analyses revealed that male smokers had lower ReHo than male non-smokers in these regions, but no differences were observed between female smokers and non-smokers (Figure 2B). The ReHo in these regions showing interaction effects between smoking and sex did not significantly differ between male and female non-smokers, whereas male smokers demonstrated less ReHo than female smokers in these regions.

**Table 3:** Brain regions showing significant smoking-by-sex interaction on regional homogeneity (ReHo) (voxel-level *p* < 0.05 corrected for false discovery rate).

**Identified brain regions**	**Peak coordinates (MNI)**			**Side**	**Peak F**	**Cluster size voxels)**
	***X***	***Y***	***Z***			
Fusiform gyrus	−36	−78	−15	L	30.35	32
Cerebellum Crus 1/Inferior temporal gyrus	−45	−48	−33	L	25.89	68
Ventral stiatum/putamen	21	15	−12	R	24.88	56

*MNI, Montreal Neurological Institute; R, right; L, left*.

**Figure 3:**
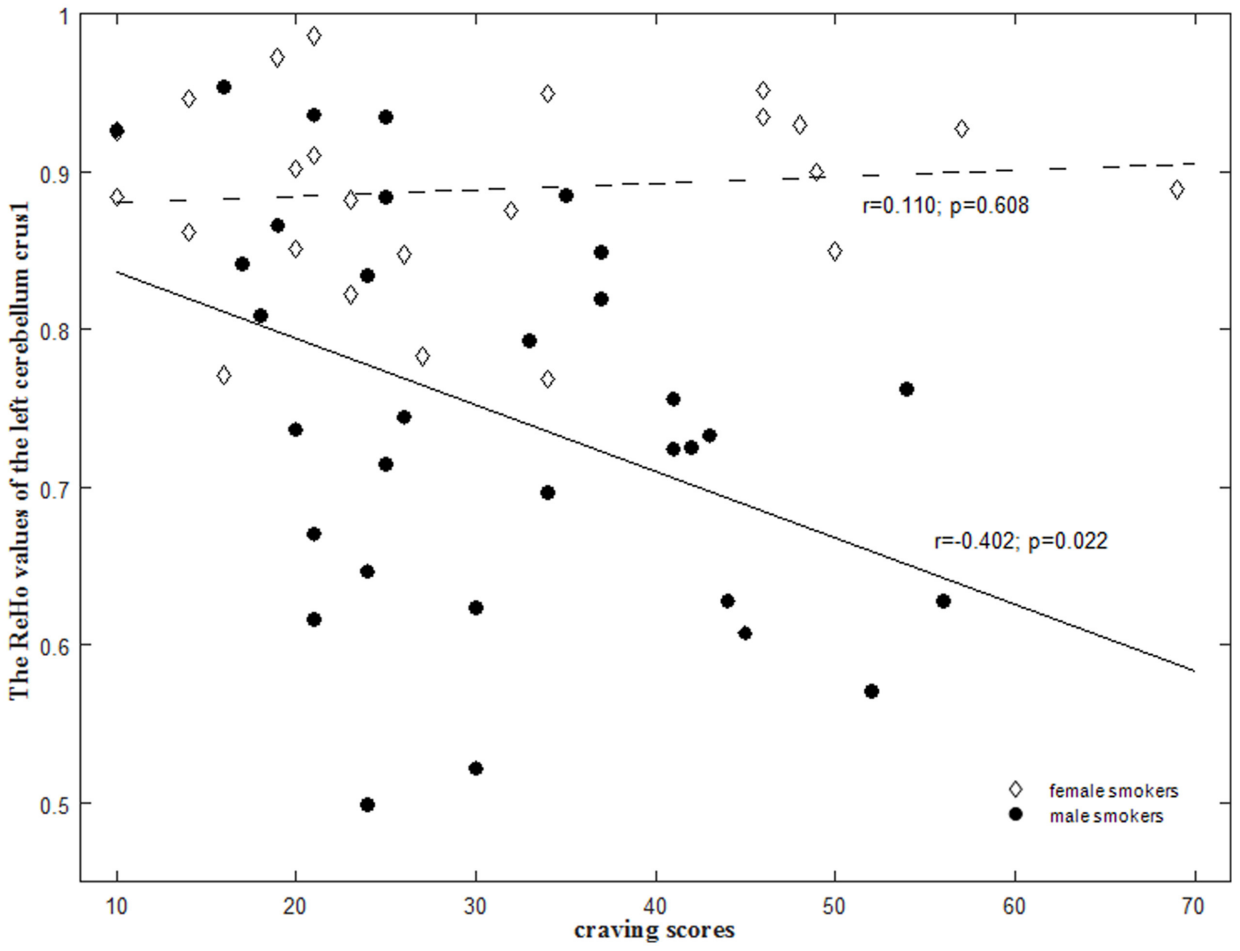
Sex-specific correlations between the regional homogeneity (ReHo) values and smoking-related measures in smokers. There were significant negative correlation of the mean ReHo values in the left cerebellum crus1 with the craving scores in male smokers (*r* = −0.402; *p* = 0.022) but not in female smokers (*r* = 0.110; *p* = 0.608). These correlations between ReHo values and craving scores were significantly different between male and female smokers (*p* = 0.03).

We also found that the lower ReHo in the left cerebellum crus1 exhibited higher craving scores in male smokers (*r* = −0.402; *p* = 0.022), whereas there was no significant correlation between the ReHo and the craving scores in female smokers (*r* = 0.110; *p* = 0.608) (Figure 3). The ReHo in the left cerebellum crus1 were more strongly correlated with craving in male smokers than in female smokers (*p* = 0.03).

## Discussion

In this study, we investigated sex-specific differences in local spontaneous brain activity between smokers and healthy non-smoking subjects using rs-fMRI and ReHo analysis. Our findings demonstrated that both male and female smokers showed higher ReHo in the left precuneus compared to sex-matched non-smokers while only male smokers had greater ReHo value in the cerebellum crus 1/2. However, male smokers had lower ReHo in the right medial OFC extending to the ventral staiatum/amygdala/parahippocampal gyrus, right lateral OFC, left cerebellum crus 1 extending to the inferior temporal gyrus, and left fusiform gyrus. Moreover, the ReHo in the left cerebellum crus1 was negatively correlated with craving scores in male smokers but not in female smokers. Taken together, these findings suggest that cigarette smoking may have differential effects on the spontaneous brain activity between men and women, especially in regions that are associated with reward and motivation functions, which are known to be affected by nicotine dependence.

One of the main results of this study using smoking by sex interaction, was found to reduce ReHo in several regions, such as the right ventral striatum/putamen, left fusiform gyrus, and left cerebellum crus 1 extending to the inferior temporal gyrus (Table 3; Figure 2). The ventral striatum is functionally implicated in reward anticipation, reward-related behaviors, and reward outcome of individuals with substance use disorders (26) and it is a reinforcement site (27). Men who smoke for the reinforcing effects of nicotine have significant responses in the ventral striatum. Using the same dataset and functional connectivity analysis, Lin et al. found that smoking has differential effects on the amygdala-OFC circuits between men and women, which is characterized by greater functional connectivity in the male circuits than that in women (12). Therefore, the change of ventral striatum activity may indicate that the reward learning defects of nicotine-addicted male individuals will eventually lead to the impairment of decision-making ability.

There is increasing evidence that cerebellum is involved in substance use disorders (28). Cerebellum was considered as a critical component of the addiction circuit, which is related to coordination of the smoking movement, motivation driving, and inhibition control (28). Male smokers, but not female smokers, have great cerebellar β2-nicotinic acetylcholine receptors (nAChRs) availability (29) and low putamen D2/D3 receptor availability (30), which may affect the up- and down-regulation. A PET studies showed that cigarette smoking increased density of nicotine binding and increased CBF in the cerebellum, which was correlated with plasma nicotine levels (31). Unlike the consensus that the GMV of cerebellum crus 1 in nicotine dependents is less than that in the controls (32–35), the activation of cerebellum varied with the expected degree of drug during exposure to drug-related cues rather than neutral cues (28, 36). In this study, we found decreased ReHo in the cerebellum crus 1/2 in male smokers (Table 3; Figure 2), while our previous studies on middle-aged heavy smokers reported that the ReHo of cerebellum increased (11). Lower ReHo in the left cerebellum crus 1 was correlated with the higher craving scores (*r* = −0.402; *p* = 0.022) in male smokers. This may be explained by the significant differences in smoking duration (SM 5.78 ± 2.84 years, SW 4.50 ± 2.50 years vs. 25.42 ± 9.10 years), and the complex mechanisms of chronic smoking, such as early dysfunction and late compensation Interestingly, female smokers did not show the same pattern of brain activity as male smokers. This finding may be partially explained by circulations in the menstrual cycle phase that modulates the reward-related regions in females (37, 38). An arterial spin labeling study examined the neural activity in response to video clip of smoking clues in females, differences emerged in the medial OFC in the follicular phase of the cycle (when progesterone levels are low), but no significant differences were observed in the luteal phase (39). Together, one may speculate that altered activity in the ventral striatum and cerebellum may indicate the selective effects of smoking on dopamine receptors and nicotinic receptors in men and women. The effect might be hampered in male smokers because of their greater reliance on nicotine. Female smokers may be exempted from smoking addiction because of their menstrual cycle.

Our team has been focusing on structural and functional MRI to study the neural mechanism underlying substance addictions. For example, Wu et al. (11) revealed that ReHo was significantly reduced in default-mode, frontoparietal attention and inhibition control networks, and regions related to motor planning increased in among the middle-aged chronic smokers (11). Lin et al. (40) and Cai et al. (35) reported the decreased nodal efficiency in default-mode and cerebellum-striatum networks, respectively, among the middle-aged chronic smokers. Qiu et al. comparing the differences of whole-brain ALFF between young adult smokers and controls, it was found that the ALFF of smoking group in the right MPFC/ventral striatum and left temporal gyrus was significantly higher than that of healthy group (41). Similarly, compared to non-smokers, our study observed that significant spontaneous brain activity changes in the right medial OFC extending to the ventral striatum, amygdala and parahippocampal gyrus of young adult smokers (Table 2; Figure 1), which is consistent with our previous results.

It is well-accepted that addiction produce the long-lasting functional and structural plasticity alterations in the corticostriatal-limbic circuitry (42). The medial OFC, ventral striatum, and amygdala, are core components of the corticostriatal-limbic circuitry related to addiction. The medial OFC is involved in integrating endogenous and exogenous information to make decision, playing an important role in the development and maintenance of addictive behaviors (43). The parahippocampal gyrus and amygdala are brain areas associated with emotional and memory processing (44), which may mediate the relief from negative emotions by smoking (45). The mesocorticolimbic dopamine systems including the amygdala, nucleus accumbens, and prefrontal cortex, are thought to be involved in burst release of dopamine and production of pleasure feelings (46). The finding of dysfunction within the prefrontal-striatal circuits is analogous to previous task-related fMRI studies showing greater activity in the reward regions in response to SCs relative to non-SCs (47–49). Similarly, rs-fMRI studies showed disrupted activity in the medial frontal cortex and precuneus/posterior cingulate cortex (11, 50, 51). Moreover, structural MRI studies exhibited that smokers had lower gray matter volumes or densities in the medial frontal cortex, precuneus, parahippocampal gyrus and temporal lobe (52–55) and reduced cortical thickness in the medial OFC (56). Taken together, our study backed up the idea that the imbalance between cognitive and reward functions in the brain may lead to smoking addiction.

Several limitations in this study must be considered before interpreting the findings. First, the number of participants in each group is relatively small, which may bias the sex-specific effects of cigarette smoking on spontaneous brain activity. Second, age or education level was not well-matched among groups. Although these factors were used as covariates in the statistical analysis, potential effects from these factors cannot be absolutely ruled out. Future studies with matched larger samples should be needed to verify the results. Third, this study is staged, longitudinal study is needed to draw conclusions about whether sex differences are a cause or a consequence of nicotine addiction. Therefore, our findings should be considered as preliminary and further longitudinal studies with larger sample size should be investigated to formulate final conclusions.

In summary, the harm of cigarette smoking is recessive, and to a certain extent it is lagging. It is crucial to investigate the different neural mechanisms underlying male and female in smoking behaviors and nicotine dependence. Our findings indicate that cigarette smoking has differential effects on reward-related activity between men and women. Our study may provide a methodological framework for education of quitting smoke, intervention treatment, and health management.

## Data Availability Statement

The raw data supporting the conclusions of this article will be made available by the authors, without undue reservation.

## Ethics Statement

The studies involving human participants were reviewed and approved by the Research Ethics Committee of Renji Hospital, School of Medicine of Shanghai Jiaotong University. The patients/participants provided their written informed consent to participate in this study.

## Author Contributions

FL and HL designed and conceptualized the study. XH, YW, WD, YS, and YZ collected the clinical and MRI data. YK organized the data. ZW and FL analyzed the MRI data and wrote the manuscript. All authors contributed to the article and approved the submitted version.

## Funding

This work was supported by the National Natural Science Foundation of China (Grant Nos. 81571757, 82171885) and the Frontier Scientific Significant Breakthrough Project of CAS (Grant No. QYZDB-SSW-SLH046), the Shanghai Science and Technology Committee Project (Natural Science Funding; grant number. 20ZR1433200), and the Explorer Project in Shanghai (Grant No. 21TS1400700).

## Conflict of Interest

The authors declare that the research was conducted in the absence of any commercial or financial relationships that could be construed as a potential conflict of interest.

## Publisher's Note

All claims expressed in this article are solely those of the authors and do not necessarily represent those of their affiliated organizations, or those of the publisher, the editors and the reviewers. Any product that may be evaluated in this article, or claim that may be made by its manufacturer, is not guaranteed or endorsed by the publisher.
